# Consultations’ demand for a hospital palliative care unit: how to increase appropriateness? Implementing and evaluating a multicomponent educational intervention aimed at increase palliative care complexity perception skill

**DOI:** 10.1186/s12904-022-00968-7

**Published:** 2022-05-26

**Authors:** Silvia Tanzi, Gianfranco Martucci, Cristina Autelitano, Sara Alquati, Carlo Peruselli, Giovanna Artioli

**Affiliations:** 1 Palliative Care Unit, Azienda USL-IRCCS Reggio Emilia, Reggio Emilia, Italy; 2 Local Program of Palliative Care, AUSL Modena, Modena, Italy; 3 Italian Society of Palliative Care, Milan, Italy

**Keywords:** Palliative care, Complexity, Tumour board, Multidisciplinary discussions, Care pathway, Oncology, Hospital, Training

## Abstract

**Background:**

Planned, multidisciplinary teams’ discussions of cases are common in cancer care, but their impact on patients’ outcome is not always clear. Palliative care (PC) needs might emerge long before the last weeks of life. Many palliative care patients could be managed from the usual care staff, if appropriately trained; specialist palliative care should be provided to patients with more complex needs. Staff needs adequate training, so that only patients presenting a higher complexity are properly referred to the second level (“specialized”) PC services. In the considered hospital setting, “tumour boards” (multidisciplinary discussions) refer often to a low number of patients. Overall complexity of patients’ needs is hardly considered.

**Methods:**

A mixed method pilot study with data triangulation of professionals’ interviews and an independently structured evaluation of complexity of referred patients, before and after the intervention, using the PALCOM instrument. We trained four teams of professionals to deliver first-level palliation and to refer patients with complex needs detected in multidisciplinary discussions. A multicomponent, first level PC educational intervention, including information technology’s adaptation, a training course, and bedside training was offered from the specialized PC Services, to all the HPs involved in multidisciplinary pancreas, lung, ovarian, and liver tumour boards.

**Results:**

While the level of complexity of referred patients did not increase, trainees seemed to develop a better understanding of palliative care and a higher sensitivity to palliative needs. The number of referred patients increased, but patients’ complexity did not. Qualitative data showed that professionals seemed to be more aware of the complexity of PC needs. A “meaning shift” was perceived, specifically on the referral process (e.g., “when” and “for what” referring to specialist PC) and on the teams’ increased focus on patients’ needs. The training, positively received, was adapted to trainees’ needs and observations that led also to organizational modifications.

**Conclusions:**

Our multicomponent intervention positively impacted the number of referrals but not the patients’ complexity (measured with the PALCOM instrument). Hospital staff does not easily recognize that patients may have PC needs significantly earlier than at the end of life.

**Supplementary Information:**

The online version contains supplementary material available at 10.1186/s12904-022-00968-7.

## Background

Palliative care (PC) is an approach that improves the quality of life of patients and their families affected by a life-limiting disease. PC constitutes an evolving field with extensive studies demonstrating benefits for patients, families, and the healthcare system [1]. PC intervention is generally recommended earlier than it has been in the past for patients [2–4] and their caregivers [4, 5] up to what is now defined as “early PC interventions” [4, 6, 7].

Some studies recognize that multidisciplinary teamwork promotes integration between medical specialists and palliative care teams [8–10] in activating shared care models. Currently multidisciplinary care models have been developed for all care settings but specifically for the hospital setting [11, 12].

There are several studies [8, 9, 13–15] that propose the inclusion of the palliative care team in the multidisciplinary discussions of cases and in the care pathways to allow for the beginning of care of these patients as easy and early as possible. Multidisciplinary team meetings provide an opportunity to discuss patient management between the medical specialist team and the palliative care team, with the primary goal of developing shared care [16].

This integration contributes to developing the model of ‘shared care’ between non-specialist PC professionals (so-called first level) and PC specialized professionals (level II) [16, 17] as indicated below:

- First Level is based on the skills and knowledge that all professionals should possess to identify and respond to less complex palliative care needs (eg first response to symptoms control)
- Second Level, i.e., the specialist level, is activated when there are higher complex needs, regarding both the physical, relational, social, and spiritual aspects of the patient and his/her family (eg conflicting families or use of methadone for pain). The advice of a team specialized in the management of these complex situations can provide an adequate response to the needs and contribute to the growth of First Level’s skills [18].

However, the use of standardized and rigid protocols may limit the assessment of the complex bio-psycho-socio-existential needs of advanced cancer patients and their families [19].

These needs have hardly found space in the multidisciplinary discussion conducted by professionals specialized in other disciplines, who are often not used to recognizing the specific needs of palliative care. Attention to these domains of personal needs contributes to defining the complexity of the single sick person, to which one must respond with an approach that includes, when appropriate, a provision of palliative care simultaneously with active care from the team that provides the usual care of that patient.

Moreover, hospitals have a major role to play in the delivery of non-specialist palliative care [20]. Healthcare providers working in hospitals have significant exposure to patients with palliative care needs that are mostly manageable with non-specialist interventions.

A “shared vocabulary” on palliative care is essential to propose a delivery model based on recognition of patients’ needs, which also values the specialist’s decision and in the direction of personalization of care than in that of standardization.

Training for the specialists of the First Level by PCTs specialists of the Second Level becomes essential [21]. In the considered hospital setting, “tumour boards” (multiprofessional and multidisciplinary discussions, where both different professionals, as nurses and medical doctors, and different specialists, as radiologists, surgeons and oncologists, were involved) often have a low number of referred patients, usually on a clinical staging basis (e.g., metastasis presence). The level of overall complexity of patients’ needs is hardly considered, possibly representing a problem of sustainability in the future. Multidisciplinary discussions may be the right place in order to improve HPs’ PC competences.

### Aim of the study

The aim of the study is to pilot and evaluate a training course in PC complexity for health professionals (HP) belonging to tumour boards/multidisciplinary discussion group and its impact on appropriateness of referral to second-level palliative care, particularly the complexity of referred patients (which is expected to result in a higher level). The training course aimed at increasing the ability of the clinical staff to assess complex, multidimensional palliative care needs according to the PALCOM instrument (see details in the “Methods “section), and consequently to be able to refer the most demanding cases, from a multidimensional (and not merely clinical) perspective, to our Specialized Palliative Care Unit.

To our knowledge, there are no published experiences in scientific literature about training programs for operators that have the main objective of enhancement and early recognition of palliative care needs in the tumour boards/multidisciplinary discussions, in order to facilitate appropriate delivery of the specialized palliative care team for patients with more complex needs, assessed both by quantitative and qualitative methods.

## Methods

This is a Phase 0-II, mixed-method study, developed accordingly to the “MRC framework for the assessment of complex interventions” [22, 23]. As expected by this model, the process of development and evaluation of complex interventions are conceptualized in several distinct phases. Even if these phases are developed in analogy to the sequential phases of drug development, this may be seen as a more iterative process. Preliminary work is performed with the aim of highlighting the probable active components of the intervention and then deliver them effectively during the trial. The required identification of stage of development and outcome measures of the project helps providing to researchers and funding bodies a reasonable quantity of evidence that an appropriately designed study has been performed.

The study was subdivided into 3 phases.

### Phases of the project

#### Phase 1: developing the training programme

The programme was inspired by our previous experiences [21, 24]. We also reviewed the literature searching for systematic reviews on existing Palliative Care training programmes, focusing specifically on tumour board and/or multidisciplinary oncology discussions but no previous experiences were available.

Potentially eligible physicians for the training were interviewed, gathering information on their perception of educational needs in this field, and the programme was developed accordingly. We then performed qualitative analysis using thematic analysis (see “Data collection and analysis” section).

#### Phase 2: assessing the quality of the implementation

This phase was aimed assessing the consistency of the implementation process. Thus, information on the achievement of the expected goals was collected for each component of the programme (Table 1).

**Table 1:** Complex intervention components and their results

Components	Realization
*Information technology design adaptation* • Introduced Field for PC physicians’ presence • Introduced Field for PC necessity to referral	As planned
*The PC needs assessment* • 1 FG for each multidisciplinary group before the training course	As planned for hepatocarcinoma group and ovarian cancer group Professionals’ single interviews for lung and pancreatic cancer group
*The training course* (3 theoretical lessons in 3 afternoons) • Assessing the physical, psychological, social, and spiritual symptoms. • Breaking bad news to patients and families • Sharing decision making with patients and families	As planned for lung and hepatocarcinoma group; A single, 6 hours-long-day for pancreatic and ovarian cancer
*Bed side training* *(the pc consultations performed after the training in the departments where trainees daily work)* PC Consultations realized 3 months before and 3 months after the training course	As planned
*Trainees’ evaluation* 1 Focus Group after the training course for each group	As planned for hepatocarcinoma group and pancreatic group Single interviews for lung cancer professionals For ovarian cancer the evaluation was interrupted for Pandemic
*Patients’ evaluation* By PALCOM tool 20 patients before and 20 patients after the 3 months previous and 3 months after the training course	See Table 2 for details

#### Phase 3: assessing feasibility and implementation methods

During this phase, the feasibility of the implementation process within the hospital setting was assessed.

Both the procedure and the intervention were implemented through a convenience sample of 4 tumour boards, to assess the quality of the implementation.

We considered the programme feasible if:

- the different components of the training course were identified
- the programme was delivered as established to the 4 groups.

A mixed-method, evaluation study was performed. In fact, we triangulated qualitative and quantitative collections of data from the same time frame, in one separate analysis.The outcomes, measured by number of referred patients and their level of complexity assessed with the PALCOM instrument, were compared [25–27]. The PALCOM is “An ad hoc structured evaluation including socio-demographic and clinical data, symptom burden, functional and cognitive status, psychosocial problems, and existential-ethic dilemmas […]. According to this multidimensional evaluation researchers can classify patients “as high, medium, or low palliative complexity, associated to need of basic or specialized PC” [28].

The Moore model was the evaluation framework of this study project. It consists of five orders of learning, from Attendance (Level 1) to Change in Practice Performance (Level 5) [25]. The model is routinely used in the continuing medical education program (CME). The fifth level (change in performance) has been assessed in two different ways (see Tables 2, 3 and “Phase three” paragraph in the Results section): quantitatively (number of overall referrals to palliative care services) and qualitatively (post-intervention interviews).

**Table 2:** Quantitative results on Patients complexity

PALCOM Complexity score	Low PRE (3 months)	Median PRE	High PRE	Low POST	Median POST	High POST
**Lung tumourboard** **(pts addressed to PC service, number)**	**13**	**4**	**0**	**3**	**1**	**0**
**Pancreatic tumourboard** **(pts addressed to PC service, number)**	**4**	**2**	**0**	**1**	**0**	**0**
**Hepatic tumourboard** **(pts addressed to PC service, number)**	**3**	**0**	**0**	**0**	**0**	**0**
**Ovarian tumourboard** **(pts addressed to PC service, number)**	**0**	**0**	**0**	COVID-19 period	COVID-19 period	COVID-19 period

**Table 3:** Meaning shift in themes before and after training

THEMES	BEFORE THE TRAINING	AFTER THE TRAINING
Multidisciplinary groups’ structure	Focus on professionals’ functions	Focus on patients’ needs
When I level should call PC team	Very different opinions	Difficulty of the palliative approach
For what needs PC team is required	Specific problems	High complexity patients
Pertinence of requests for pc intervention	We do not know	Two proposals to evaluate pertinence

The study aimed to evaluate, both quantitatively and qualitatively, the impact of the training related to:

- Increasing of Palliative Care competencies regarding complexity (Moore Level 3);
- Evaluation in terms of participants’ performances addressing more complex palliative care patients to Specialized PC Service (Moore Levels 4 and 5).

Revised standards for quality improvement in reporting excellence (SQUIRE 2.0) [29] were applied to draft this manuscript.

### Context and sampling

The General Hospital “Arcispedale Santa Maria Nuova”has a Specialized Palliative Care Service (SPCS). It provides specialist care both in the 900-bed facility, insignated with the “OECI” title (Clinical Cancer Centre by the Organization of European Cancer Institutes). The SPCS is active since April 2013 and currently includes seven members, three physicians and two nurses with a significant experience in the field, and a nurse and a physician dedicated to research and education. SPCS is specialized in performing training programs, research in palliative care trainings, and quality improvement programs [21, 24, 30, 31].

The trainees were physicians and nurses of HPs of the Hepatocarcinoma tumour board, Pancreatic cancer tumour board, Ovarian cancer tumour board, and Lung cancer tumour board. Weekly or bi-monthly tumour boards were organized during the year. Standards for staging evaluations, medical imaging, chemotherapy choices, radiation therapy, and surgical approach were routinely discussed. Specific indicators established with the Head Department (eg time spent from diagnosis to surgery, days from diagnosis to chemotherapy and so on) were tracked annually. HPs discussed all new diagnoses of cancer and decided on the care pathways (including surgery, chemotherapy, radiotherapy, addressing to pc service) of single patients.

Consequently, patients analysed by this study were advanced lung cancer patients, advanced pancreatic cancer patients, hepatocarcinoma patients and ovarian cancer patients.

### The intervention

#### IT design adaptation

In order to design the informational system as easy use for the staff [32], an optional yes/no field was set up so that the physician presenting the case to the board could signal the necessity of the presence of the palliative physician at the discussion. Another optional yes/no “activation of palliative care” field was also introduced in the discussion, so that the potential board’s decision of activating palliative care could be clearly recognizable. This field was also used to identify the patients eligible for pre−/post- training complexity evaluations reported in this study to assess the impact of the intervention.

#### Training program

The training program (Table 4) for each tumour board was initially planned to last for 12 hours in 3 afternoons. Three macro themes, one for each afternoon, were identified, according to a largely approved model of palliative care approach [18]:

- Assessing the physical, psychological, social, and spiritual symptoms. PROMS used in clinical practice were taught (IPOS eg)
- Breaking bad news to patients and families
- Sharing decision making with patients and families

**Table 4:** Training participant’s characteristics

	Sex (M/F)	Age (median)	Professions (Physicians/Nurses; all participants were post-graduate level)
Lung cancer group	3 M 5 F	42	4 physicians 4 nurses
Pancreatic cancer group	3 M 6 F	46	8 physicians 1 nurse
Hepatocarcinoma group	2 M 5 F	51	4 physicians 1 nurse 2 technicians
Ovarian cancer group	2 M 7 F	44	6 physicians 3 nurses

Other specific training needs were analysed from the focus groups.

#### Bedside training

**Fig. 1:**
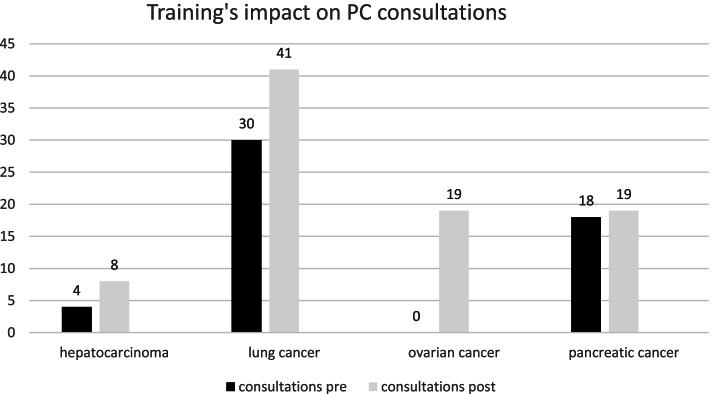
Impact on PC consultations numbers

The theoretical lessons were completed with specialized consultations at the bed side; PC specialists performed several consultations in the department where trainees work daily (see Fig. 1) in the months after the training.

### Data collection and analysis

#### Phase I

- Educational needs of the interviewed professionals were detected through the qualitative analysis of the interviews performed before the intervention, that were recorded and transcribed *verbatim*. The researcher encouraged interaction between the participants and elicited a range of opinions/views starting from a discussion of a case study that was specifically designed for the intervention [33]. The qualitative analysis was performed independently by Two researchers (AG and GM), who read the interviews, tagged them and categorized them into themes. The final categorization was determined through further discussion between researchers, addressing any relevant disagreement in their analysis.

#### Phase II

- The pilot implementation process was analysed, determining an overview of the objectives achieved and not achieved for each component of the programme. (Table 1).

#### Phase III

For each multidisciplinary group, one FG after the training were planned. A moderator and an observer were present during every FG session.

The qualitative analysis adopted the framework method described by Gale et al. [34]. The analysis concentrated on emerging themes, with a specific focus on possible changes in the way the professionals see palliative care and palliative care needs. This allowed for the search of any possible changes in meanings [21] from before to after the training. In order to increase trustworthiness, the overall process was conducted independently by two different researchers, GM and GA, that started assigning tags and categories, then identifying the main themes emerging, and any divergence was supervised by a third researcher (ST). The “Atlas TI” software was used to categorize the dataset.

In reference to the quantitative assessment, patients addressed to palliative care service were analysed 3 months prior and 3 months after the training course using PALCOM as the framework [28]. Italian validation of the whole instrument was not available, but singular components were already in use in clinical practice. It was composed of 5 well-known domains, measured with instruments that have been individually validated in Italian. These instruments were the performance status by Karnofsky, the physical and psychological symptoms assessment by ESAS, the Edmonton staging system for cancer pain, a checklist of general socio-familiar risk factors and a list of specific ethics topics. The difference in the PALCOM results before and after the training course were analysed, and the number of PC consultations before and after the intervention were registered.

We performed data triangulation through quantitative and qualitative results comparison [26] and through a thematic analysis of qualitative data performed independently by two researchers and the comparison of their results, to a consensus through the supervision of a third researcher.

## Results

This study was focused on the development of a training program and its preliminary assessments.

Table 1 summarized the main components of the intervention and if they were achieved as planned.

### Phase 1: developing the training programme

#### The focus group and interviews with professionals

A convenient sample of health professionals participating to the tumour boards were interviewed in the 6 months before the implementation of the training course to collect their perceived educational needs and tailor the contents of the course to accordingly. One focus group was conducted for every tumour board. In the lung and pancreatic tumour board we conducted multiple single interviews instead of focus groups after multiple failed attempts, due to difficulties of the considered staff to be present at the same time. The interviewees gave useful suggestion on the topics and the methodologies that they felt more interesting and appropriate for them. According to their preferences, we rearranged the timetable of the course and focused more on experiential aspects of training, as the discussion of complex real cases (see the “Qualitative results” section, Table 1 and discussion for further details).

##### The training programme

Considering both the recognized PC model and the difficulties that the professionals presented during the interviews, we developed the training program.

The key features of the programme were the following:

- the programme was implemented within the hospital multidisciplinary groups, i.e., in the context in which participants are required to practice PC skills that they are learning.
- the hospital SPCS conducted the training programme,supported by professionals with a psychosocial background, such as psychologists or counsellors.
- The program evaluation was based both on trainees’ knowledge changes and the complexity of referred patients as established by PALCOM tool.

The programme aims at improving physicians’ competencies in the following three major areas: 1) exploring social psychological and physical patients’ needs, 2) practice difficult conversation with patients, and 3) sharing decision making with patients and families.

### Phase 2: quality assessment of the programme

As required in the MRC framework, we assessed if and how the different components of the programme were implemented, as described in Table 1.

### Phase 3: preliminary assessment of the programme: the evaluation system

#### Quantitative results

There were no conclusive data on possible differences in complexity in patients discussed during the tumour board and in reference to PC before/after the intervention (Table 2).

The number of patients with a high level of complexity according to the PALCOM complexity score didn’t see any increase after the training. During the training period the numbers of PC consultations for both inpatients and outpatients for hepatic cancer, lung cancer and ovarian cancer improved as shown in Fig. 1. Consultations were similar for pancreatic cancer. In any case, the numbers were still too low to be statistically significant and result any possible causal attribution.

#### Qualitative results

The program was established for two out of four multidisciplinary groups with three theoretical lessons on three themes. SPCS, as requested by the team of trainees, changed the training in a single lesson of 6 hours for pancreatic cancer team and ovarian cancer team. Only the Hepatocarcinoma team participated in the two planned focus groups: one for pre-training and one for post-training. The FGs were organised in co-occurrence of the weekly meetings to optimize the professionals’ availabilities. Despite various efforts, the other teams were only available for one focus group and some semi-structured interviews of single professionals. In spite of the good feedback from the trainees, many changes to the initial plan were necessary to ensure the feasibility of the intervention.

By analyzing the contents of the FGs and interviews as a whole, a *meaning shift* is highlighted as summarized in Table 3.

### Multidisciplinary groups’ structure

(from “a focus on professionals’ functions” to “a focus on patients’ needs”)

#### Focus on professionals’ functions

Before training, within the multi-professional groups, every specialty had its own perspective: some integrated and others in addition to the others. Different opinions coexisted: some physicians argued that it would be important to have all the clinicians see the patient before such a decision as palliation could be made. Others suggested that even if the interprofessional group formally existed, in some aspects, each professional performed his specific function which was simply added to the others.

*“Now, the doctor who has to take in charge the patient visits the patient first, because he needs to be seen by the surgeon or the oncologist, or any other professionals. He needs to examine him and then they decide all together, but the patient must be seen, it cannot be a decision based on a single doctor’s idea” (cod. 3.4)*

*"“We (clinicians) treat the local site, surgeon does the surgery… when we discover that nodules are many, or if we can’t follow them up anymore, we leave them directly to the oncologist, for what we could call a pre-palliative stage, of palliation almost”.." (cod. 1.41)*

#### Focus on patients’ needs

After the training, the professionals reported that PC has a curative and active role but also recognized that it has a prominent role in listening to patients’ needs, which is understood as different from the needs of the disease.

Some participants stated:

*“Now, at the end, if you could manage to listen a little bit more to the patients, you could understand different needs from the ones related to disease” (cod. 2.88)*

### When to call PC team

(from “Very different opinions’ to “Difficulty of the palliative approach”)

#### Very different opinions

Before the training, different opinions were highlighted. According to some doctors, all patients, family members, and professionals could take advantage of the palliative’s consultations. For others, palliation was mainly about pain management, while some perceived that in their ward the palliative care team was activated only in extreme cases. The professionals expressed themselves by:

*Since we discuss 200 patients in a year, theoretically almost all the patients might be [included]… both patients and families, but also ourselves, benefit from an interview with palliativists to better manage the path of patients… it’s unthinkable, I mean, to activate all this tide of consultations”* (cod. 1.106)

*We go to the opposite extreme, we activate the palliativists only when situations are desperate” (cod. 1.107)*

#### Difficulty of the palliative approach

After the training, the multidisciplinary team had sharper ideas on which skills are involved in a PC intervention but still thought that such an intervention could be strongly related to the sensitivity of the individual doctor, being a very specific and somewhat difficult intervention to evaluate, especially in a multidisciplinary discussion that is usually focused more on early stadiation and lines of chemotherapy than on a real multidimensional evaluation of the advanced patient.

*" It is already enough that… the team discusses mostly path and treatment management. Palliative care is a treatment too, and so is the taking charge of the patient, but it’s something… so specific, and with a sensibility which differs from doctor to doctor, without seeing the patient and without knowing his clinical and familiar story and everything… it’s difficult to understand, during a multidisciplinary discussion.” (cod. 5.2)*

### Needs that require the palliative care team

(from “Specific problems” to “High complexity patients”)

#### Specific problems

Before the training, the professionals identified individual problems for which to request the advice of the PC team. In particular the problems included: the communication of the diagnosis and poor prognosis; the lack of support from family and caregivers; and the end of life during which professionals no longer knew what to do. The professionals said:

*“Therefore, sometimes there’s the problem of how much information should be given to the patient and to the relative. To the patient, generally we communicate everything because we have to put him through the procedures, so it’s really important that they understand…” (cod. 1.92)*

*“…when the situation gets worse, family members, even the ones that we haven’t seen until then, they come and ask me why we are in a so progressed stage… it’s a considerable difficulty because you can talk, you can write as much you want but the message doesn’t arrive…” (cod. 1.54)*

*“For us It’s the care phase, when patient can’t manage it anymore, he often rings the bell (…) or he comes to do the paracentesis and he asks ‘Why am I like this? Am I going to make it? Why I feel so sick?’ (cod. 1.115)*

*We don’t know how to answer… sometimes we are speechless… we don’t know what to tell the patient” (cod. 1.116)*

#### ‘High complexity patients’

After the training, the professionals did not report any single problem, but they recognized the patients’ subjectivity and the complexity of the situation with all its possible perspectives. They went from the problem of prognosis to the need to accompany a patient in a progressing disease, or the management of a patient who denies the illness. The focus was not the disease, but the patient with an uncollaborative family or very anxious traits.

*“He was a young patient, with an advance stage hepatocarcinoma (…) we thought to contact people close to his family environment to create around him a safety net, otherwise when he feels sick he had to manage all by himself” (cod. 2,22.36)*

*“Because [the patient] he’s so anxious and if I go to tell him ‘We want to start palliative care’ it might become a sort of tragedy (…) I didn’t propose him palliative care yet because, maybe, now he’s not able to accept it, so I need to find the right moment, because he’s very fragile…” (cod. 2,39. 52)*

### Pertinence of requests for PC intervention

(from “We don’t know...” to “Two proposals to evaluate pertinence”‘)

#### We don’t know..

The pertinence theme of PCT involvement was already present in the FG before the training. Professionals questioned the pertinence of their requests to the PCT, recognizing that making appropriate requests became important in providing effective responses to patients and their families. Sometimes they learned from mistakes as well.

In fact, this was how the professionals expressed themselves:

*“I mean, [we need] ideas to understand if we have to intervene before and how, and what is the right moment to include the palliative doctor, of course yes, because we all have to learn, myself first…”* (cod. 7.0)

*“I would like to know from them [the palliative care physicians] how some referrals happen to be ‘inappropriate.’ I mean, which are the criteria that make a request become inappropriate?”* (cod. 6.9)

*“I mean ‘You have to call us earlier, so that we can avoid the situation where we see the patient 3 days before death’ (the palliativists said to us); It might be for this…”* (cod. 6.8)

#### ‘Two proposals to evaluate pertinence’

After attending the course, the initial awareness of pertinence was followed by a more proactive phase. The multi-professional groups identified two possible proposals to improve integration with the PCT:

- Within the multidisciplinary meetings, they proposed to ask themselves a question: “*Are the PCs or the activation of the PCs necessary?”*
- They asked for future retraining and a more frequent feedback from the palliativists, after the patient has been sent for consultation, to understand if the reports to the PC have been appropriate or not. Professionals claimed:

*“Should we ask ourselves “Are palliative cares, or the activation of the palliative care team, necessary? I mean, to us… it’s useful to understand: is the referral really needed? I mean, did we miss something?”* (cod. 6.2)

*“They should provide us a feedback and say ‘Okay, you have discussed 10 patients, but for this and that, you should have called the palliative care service’; in this way we could understand if the problem was ours or if it was someone else’s/..” (cod.6.3)*

### Comparing of qualitative and quantitative results

The participants were very active and participatory throughout the training programme and research: they all attended the course from the beginning to the end; they participated at the evaluation at the pre- and post- qualitative evaluations. Qualitative and quantitative data converge in some aspects and diverge in others.

The total amount of referrals increased in two out of three of the wards involved in the training, and the qualitative results suggest a deeper understanding of the palliative care role and advantages.

On the contrary, we cannot see any significant increase in the complexity of the patients referred to the PC service, when comparing the measurements of the PALCOM instrument.

## Discussion

Considering the aim of the study, we can say that HPs have shown, from a qualitative point of view, an increased capacity to see complexity. However, from a quantitative point of view, the patients referred to the SPCS after the intervention were not more complex patients after comparing the PALCOM evaluations of pre- and post-intervention referrals.

The training course’s initial plan needed rearrangements in some cases, e.g., as lessons were reorganized according to the tumour board participants’ requests provided during the assessment of educational needs, in order to offer a more trainee-centred education. Some clinicians suggested to increase the experiential part of the learning experience, such as roleplays and cases’ discussions, at the expense of the theorical part. The quantitative evaluation of the course has shown an improvement of the number of PC consultations requested in two of the three groups that were trained and had the chance to continue with the planned clinical practice, while the other trained groups found it impossible to acquire data due to the first wave of the COVID-19 pandemic in Italy that suspended the usual network of care. Some suggestions by trainees to improve PC competences were provided by participants, such as: having feedback from PC team on referred patients (as also suggested by evidence from implementation research [35, 36], repeating PC training routinely, and having a PC specialist on demand for more complex cases.

In literature, the first studies on complexity in PC have shown a percentage of advanced complex cancer patients from 40 to 50% in total number of advanced cancer patients examined [28]. Nonetheless, this number is far from the patients’ number referred to our service in both the 3 months before and the 3 months after the training course. Interviews confirmed that the tumour board discussions on patients were predominantly based on imaging and chemo-radiation treatment or surgery, with marginal focus on patients as a whole (comprising symptoms and psychosocial/spiritual issues). Moreover, interviews suggested that multidisciplinary meetings tended to focus more on patients that were in the diagnosis-related stage, while complexity was perceived usually later, during the more advanced treatment stage.

From our interviews and FG, some participants of the tumour boards discussions perceived their role in recognizing complexity as marginally relevant, due to a more professional focus on technology (e.g., endoscopies and radiation treatments). They did not think of themselves as the right professionals to adequately assess PC consultation need for the patients.

Despite the failure to intercept more complex patients, the number of in- and out-patients PC consultations requests improved. This suggested that a major sensitivity from the trainees to the SPCS role was achieved. Those professionals usually participate and worked in the tumour boards, such as in the gynaecological and oncologic surgery department for the ovarian cancer tumour board, the respirology department for the lung cancer tumour board, the infectious disease unit and internal medicine department for hepatocarcinoma tumour board. From this reasoning, it is likely that the consultations from these departments improved, as supported by the qualitative retrieval of a “meaning shift” in the reported perception of the role of the board itself and the process of referral (see “Results” section).

The need for education in palliative care was largely advocated by healthcare providers, and lack of specific training opportunities in palliative care principles was highlighted.

The literature contains several barriers, including cognitive barriers, that can delay the integration of PC and efforts to promote a collaborative approach: a lack of awareness of palliative care, collaboration and communication in contexts related to palliative care, differing attitudes and beliefs towards palliative care and emotions involved in the pathways of the disease [37, 38]. According to the study, when participants talk about the concept of *Palliative Care,* they present different conceptualizations. Some understand PC as a way of managing the psychopharmacological aspect of end-of-life patients, while others understood it as a possible access to specific psychological support. Some participants also identified PC as pain treatment, while others understood that this is not the exact role of PC. Some considered PC consultation as a way of reducing the need for requesting internal consultations in the dying patients. These different points of view delay the referral to PC service as described in a recent review by Nevin et al.: ^“^Although the benefits to applying principles of palliative care early in disease trajectories is well established, specialist versus non-specialist palliative care is contentious at times and can lead to role confusion and missed opportunities to provide effective palliative care” [39].

The review attempted to gain a deeper understanding of the unique perspectives of non-specialist PC in the hospital setting through a qualitative systematic review and thematic synthesis. The review confirms how palliative care understanding has subjective differences and varies between care providers. Furthermore, many healthcare providers described a lack of clarity on a clear definition of non-specialist palliative care and were perceived as frustrated due to the lack of clarity in their role in non-specialist palliative care provision: as suggested also by Finucane et al., “Uncertainty around what complex needs are and ambivalence regarding the hospice services available are features of the current system” (40).

Participants have “qualitatively” increased their understanding of the complexity in PC, but still refer to patients with the same level of complexity as before the training and do not recognize higher levels of complexity during the tumour board sessions. The theoretical recommendations in this field are far from what is practiced in the real-world scenario; as such the review is in agreement with the results.

Trained colleagues are assumed to achieve a higher sensibility for PC consultations, but not a higher capacity to practically assess complexity. However, from a qualitative point of view, the training project raised awareness of the complexity of PC needs, as reported from their interviews. The colleagues expressed positive opinions on the training, saying that “both technical and relational training made it possible to respond and manage patients’ outbursts.”

The presence of a PC team at multidisciplinary discussions is not perceived as essential, even if its intervention is much valued from the professionals.

### Limits

While the quantitative impact of the course can be assessed, the dimensions of the educational impact of the learning process on the learners were not due to the fact that pre- and post-training interviewees were not the same people in all interviews or focus groups. A deeper assessment on the training’s impact on learning would have been possible only by interviewing the same participants before and after the course.

## Conclusions

While our pilot multi-component intervention failed to induce a PALCOM-measurable difference in referred patients, it achieved a higher comprehension of the patients; needs complexity and of referred specialist palliative care in trained professionals, as well as positively impacting the overall number of referrals from the trained staff.

The intervention also provided useful organizational suggestions from the trained staff itself that have been incorporated in the patients’ pathways.

In fact, the intervention elicited new adjustments to the clinical care pathway of the tumour boards. Despite the major challenge requested for a hypothetical next training program, unexpected relevant secondary outcomes were achieved, as a new indicator was introduced in the clinical pathway regarding to annual meetings with feedback on patients as requested by professionals to the SPCS.

Furthermore, the Infectious Disease Unit, whose head of the department participates in the hepatocarcinoma tumour board, requested a prolonged training to SPCS on communication, symptoms control and staff support which will be implemented next year for the entire department.

A possible scenario that must be considered include a structured use of instruments to assess the complexity of needs, as the PALCOM itself, by the tumour board professionals.

The presence of a SPCS in the hospital performing out-patients visits and consultations is plausible with the chance of sharing complex cases with colleagues during their daily work. This remains as one of the most relevant pathways to teach complexity.

## Supplementary Information

**Additional file 1.**

## Data Availability

The datasets used and/or analysed during the current study are available from the corresponding author on reasonable request.

## Abbreviations

PC: Palliative Care
SPCS: Specialized Palliative Care Service
IT: Information Technology
HP: Health Professionals
