# Correction: Consultations’ demand for a hospital palliative care unit: how to increase appropriateness? Implementing and evaluating a multicomponent educational intervention aimed at increase palliative care complexity perception skill

**DOI:** 10.1186/s12904-022-00991-8

**Published:** 2022-06-01

**Authors:** Silvia Tanzi, Gianfranco Martucci, Cristina Autelitano, Sara Alquati, Carlo Peruselli, Giovanna Artioli

**Affiliations:** 1 Palliative Care Unit, Azienda USL-IRCCS Reggio Emilia, Reggio Emilia, Italy; 2 Local Program of Palliative Care, AUSL Modena, Modena, Italy; 3 Italian Society of Palliative Care, Milan, Italy

**Correction: BMC Palliative Care 21, 1 (2022)**

**https://doi.org/10.1186/s12904-022-00968-7**

Following the publication of the original article [1], the author reported that the given name and family name of all authors were erroneously transposed.

The author group has been updated above and the original article [1] has been corrected.
