# Supplementary material for: Consultations’ demand for a hospital palliative care unit: how to increase appropriateness? Implementing and evaluating a multicomponent educational intervention aimed at increase palliative care complexity perception skill
Source: BMC Palliat Care. 2022 May 26;21:90. doi: 10.1186/s12904-022-00968-7 (PMC9133822; doi:10.1186/s12904-022-00968-7)
Supplement: Supplementary file 1 — Additional file 1. [file 12904_2022_968_MOESM1_ESM.docx]

**Guide for conducting focus groups with professionals participating to the tumor boards**

**Focus groups PRE-training intervention**

**Opening**

1. Thanks to those present
2. The objectives and methods of conducting the meeting are presented
3. Starting of the audio-recording
4. Presentation of the facilitator and his role
5. Presentation of the observer and his role
6. Presentation of the participants

**Presentation of an explanatory case**

| A clinical case with complex palliative care needs, relative to the type of cancer discussed in the tumor board, is presented by the facilitator. |
| --- |

**Question-stimulus and discussion**

1. **Which may be the main problems in taking care of the patient in the presented case?**

*(Explore the criticalities and difficulties perceived by the participants in assisting cancer patients).*

**In-depth study of clinical problems**

1. [In the case proposed to you] **In particular, what are, in your opinion, the most relevant clinical unresolved issues**

*(To stimulate the participants to focus and deepen the clinical-assistance criticalities and the underlying difficulties,)*

**3.** [always starting from the proposed case] **What were, in your opinion, the main difficulties encountered in dealing with the problems correctly? What are the questions you asked yourself?**

(Explore the difficulties perceived by the participants in finding adequate solutions to clinical problems and the ability to ask adequate questions to address the problem)

**Focus on psychosocial and existential problems**

**4.** [In the case that has been proposed to you] **Have you identified other problems, of the patient and / or of the family, not limited to clinical areas?**

*(Explore participants' abilities and / or difficulties in identifying other problems - psychological, social, existential - of patients and their families, which are closely connected with the disease)*

**5**. [always starting from the proposed case] **What were, in your opinion, the main difficulties encountered in dealing with these problems correctly? What are the questions you asked yourself?**

(Explore the difficulties perceived by the participants in finding adequate solutions to the *psychological, social, existential problems - of patients and their families, which are closely connected with the disease and the ability to ask adequate questions to address these problems)*

**Short presentation of the training course**

| The training course that will be implemented is briefly explained, which will address three main issues:   - the decision-making abilities of the participants relating to the control of symptoms and less complex physical needs and the identification of relational, social and spiritual needs of the person and family - relational skills with the patient / family member, in particular for the communication of bad news (e.g. diagnosis of incurability, complications, end of active therapies, etc.) - decision-making and ethical skills of professionals, in particular the ability to make shared decisions with the person and the family, within a multi-professional team.   Methods: 3 group meetings with interactive methodologies and field training with consultations carried out by palliative doctors |
| --- |

**discussion**

***6. How would you describe your expectations regarding this training course in palliative care?***

*(Explore the expectations, the desires, the ideas that the participants have regarding the training course on palliative care)*

***7. Would you have any proposals, suggestions, specific needs that you would like to be addressed in the course?***

*(Explore proposals, hypotheses, opinions of the participants in order to co-construct the training intervention)*

**c) Closing the meeting**

**Focus group post-intervention:**

***The previous interview guide is used again, using only questions 1-5.***
